# Exploring the inequalities experienced by health and care workforce and their bases – A scoping review protocol

**DOI:** 10.1371/journal.pone.0302175

**Published:** 2024-04-16

**Authors:** Roomi Aziz, Anuj Kapilashrami, Reza Majdzadeh

**Affiliations:** School of Health & Social Care, University of Essex, Colchester, United Kingdom; Public Library of Science, UNITED KINGDOM

## Abstract

Planning for investment in human resources for health (HRH) is critical to achieve Universal Health Coverage (UHC) and establish a sustainable health system. Informed planning warrants a better understanding of the health labour market (HLM) to tackle a variety of health and care workforce challenges: from addressing critical supply shortage, to ensuring optimal skills mix and distribution, and addressing motivation and performance challenges. Scant evidence around the overall role of socioeconomic and cultural factors like gender, race, marital status, citizenship (migrant) status, workplace hierarchy etc. in determining workforce composition, deployment, distribution, retention, un- and underemployment, sub-optimal work environments and other factors in the ‘HRH crisis’ warrants further exploration. This scoping review protocol aims to map and present the available evidence on inequalities experienced by health and care workforce, the socio-economic, cultural and other bases of these inequalities, and their outcomes/ consequences. PubMed, Web of Science, CINAHL and SCOPUS will be used to identify relevant literature. All types of published study designs in English language will be included if they discuss any inequality experienced by any category of health and care workers. Elaborate keyword categories for health and care workers and inequalities context have been developed, tested and reduced to the near-final search string. Eligible articles will be charted using the Joanna Briggs Institute checklist. The sample data extraction chart in JBI manual will be used as a basic skeleton with fields added to it to serve the needs of the scoping review. Descriptive analysis will be performed, depicting basic frequencies. While no further analysis has been advised in the JBI and PRISMA protocol, thematic analysis will be undertaken; following the Braun and Clarke’s method with some modification and open coding as suggested by Maquire and Delahunt.

## Introduction

Human Resources for Health (HRH) is one of the six building blocks of health systems [1], and referred to as the backbone of health systems [2,3]. Adequate numbers, quality and diversity of HRH are critical for achieving Sustainable Development Goals (SDG) generally and target 3.8C Universal Health Coverage (UHC), indicator 3.C.1 ‘health worker density and distribution’ specifically. HRH ascertains the effectiveness of all other inputs in a country’s health system which in turn ensures the health and viability of national and global economies [4].

Although documented earlier, global HRH crisis was prominently first highlighted in WHO’s 2006 world health report [5], where the report identified crisis level shortages in 57 countries and estimated a global deficit of 2.4 million doctors, nurses and midwives then.

HRH crisis is most often expressed as shortage of health and care workers, demand or need, using measures like HRH density per 1000 population [6,7]. However, this measure limits the understanding of HRH to just a supply problem [8,9], and while useful for understanding the overall deficits in HRH availability, is not adequate for country level-planning for sustainable workforce [10].

Due to this evidence gap and emphasis on workers’ density, globally HRH planning at the policy and programmatic levels typically stays limited to supply strategies like scaling up of training capacities and building more medical/ nursing schools [11]. This is partly because most of the available evidence on HRH crisis focuses on workforce distribution and related challenges [12–19]. This traditional policy approach fails to see other systemic and structural factors and dimensions of the health labour market (HLM) [20–25].

In order to start changing that outlook, and drive evidence-informed policy design, it is important to first map the evidence available, before advocating for integration of this evidence in developing policies or programs.

In conclusion, effective HRH planning requires a deeper understanding of the structural and systematic inequalities that health and care workers experience. To accordingly inform the discourse on HRH, this scoping review aims to systematically map literature that attempts to understand the inequalities experienced by health and care workers, their bases, their subsequent outcomes and the resulting impact on HLM and health system. This mapping will present the key concepts, research methods, theories and sources of evidence in HRH research using an inequality lens [26].

## Materials and methods

Existing directories of Prospero, Figshare and Open Science Framework (OSF) were reviewed to identify if any existing protocol for a similar study existed. In the absence of any relevant registration, a priori protocol for this scoping review was developed following the Joanna Briggs Institute (JBI) approach for scoping review steps [27], as also recommended by Cochrane [28,29]. These instructions expand upon Arksey and O’Malley’s work [30] and the protocol is guided by the Population-Concept-Context framework. This protocol is also in alignment with the Preferred Reporting Items for Systematic Reviews and Meta-Analysis Protocols (PRISMA-P) and the latest PRISMA Scoping Review Extension Checklist (PRISMA-ScR) [31–33], which will guide the organisation and structure of the review (S1 File). Since the scoping review will only be based on published data with no data being collected from human participants, ethical approval is not required.

The following processes will be applied and are described in detail below:

Stage 1: Identifying the research question
Stage 2: Identifying relevant studies
Stage 3: Study selection
Stage 4: Charting the data
Stage 5: Collating, summarising, and reporting the results.

The scoping review protocol was also pre-registered with the Open Science Framework [registration URL osf.io/ktrvm]

### Stage 1: Identifying the research question

The WHO’s 2021 HLM analytical framework has for the first time described the HRH stock and HLM mismatches not just by a) numbers (shortage or surplus); or b) skills (over or under-qualified) but also c) discrimination due to the cultural and social context of the market [11]. Following this report, the new health and care workforce guides on utilising health and care workforce data are specifically highlighting how to capture discriminatory experiences of health and care workers. However, this is still a new understanding. Preliminary searches revealed limited works, and no existing systematic or scoping review looking at the range or intersectionality of inequalities experienced by health and care workers. Withing this context, PRISMA scoping review checklist guidance was followed to formulate the review questions as follows:

- What are the kinds of inequalities experienced by health and care workers?
- What are the bases of these inequalities and what are their outcomes?

Population–concept- context (PCC) framework was used to identify the main elements and conceptualize the review question. The framework was also used to identify the relevant keywords and inform the search strategy [Table 1].

**Table 1:** PCC framework for the scoping review.

Elements	Framing
Population	Health and Care Workforce
Concept	Bases of Inequalities/ Inequities experienced by health and care workforce E.g. gender, race, ethnicity, nativity etc.
Context	Health Sector / Health Labour Market

Existing scoping and systematic reviews on either of the components (population or concept) from the PCC framework were searched in PubMed and SCOPUS to identify keywords and phrases found in the titles and abstracts of papers that were likely to be included in the scoping review. Possible synonyms and combinations of the identified search terms were collected. Additional conditions like limiting to types of studies or geography were also considered and tested but eventually not included in the final protocol. The final keywords were also shared with three subject experts for their comments, to ensure that any essential HRH or inequality-related keywords are not missed.

Two sets of keywords were developed: For the population (health and care workforce) and for the concept (inequalities within health and care workforce). For health and care workforce, WHO’s definition and elaborations were used to search for possible cadres and job categories [34,35]. These definitions and categorizations encompass a wide range of health and care workers, including physicians, nurses, midwives, community health workers, and other health professionals. Understanding that literature around health and care workforce could be generic (human resources for health or health professionals or healthcare providers) or specific (nurses or dentists or surgeons) and this was incorporated in the search strategy. Categories of health and care workers unique to different national contexts were also included e.g. auxiliaries, health aides, Accredited Social Health Activists (ASHA) workers, Community Health Extension Workers (CHEWs), Lady Health Workers (LHWs) etc. Different iterations were run to reduce keywords and customize for the final database choices.

For the concept keywords set (inequalities experienced), a range of keywords was originally included, ranging from inequalities, bias and discrimination, to bullying, victimisation, micro aggressions, racism, violence and assault. However, it was decided that the search be kept open for the expected outcomes and experiences and not locked with the key words. Therefore the final words included were around inequalities, inequities and discrimination.

### Stage 2: Identifying relevant studies

On the basis of the initial literature review, a list of relevant databases has been put together, including 20+ databases accessible through University of Essex’s registration with EBSCO. Based on the subsequent discussions and finalization of scoping review objectives, a review of literature will be performed in PubMED, CINAHL Ultimate, Web of Science and SCOPUS. In addition to this, a list of five-ten extremely relevant articles will be developed and entered into Research Rabbit App and Connected Papers App to identify closest network of relevant papers and expert authors, which will also be scoped and considered for inclusion.

Initial drafts of the search strategy have been reviewed by the Library Team at the University of Essex. Prior to finalization of the search string, multiple iterations were run with different keyword and search strategy combinations i.e. searching the concept keywords in title or abstract fields, before finalization of the root search string. The final search components (both population and concept) will be applied in the article TITLE to reduce the number of irrelevant articles.

Database-specific search string variants with the final key words and relevant index terms, Boolean operators, truncation and wildcard symbols will be developed for each of these databases.

Initially it was intended that only research after the year 2005 will be included, following the WHO’s 2006 World Health Report which was an exhaustive assessment of health and care workforce situation globally [36]. However, in the final search string no date restrictions have been placed, considering the fact that the World Health Report did not specifically frame any challenges experienced unequally by the health and care workers due to their positionality.

All published research articles in English language will be included. Papers will be excluded if they do not fit into the conceptual framework of the study, for example studying inequitable health and care workforce distribution without focusing on inequalities being experienced by health and care workers, papers focusing on health workers but not their experiences, papers focusing on inequalities being perpetuated by health and care workers or being addressed by them [Table 2].

**Table 2:** Eligibility criteria.

Criteria	Characteristics	Decision
**Language**	English Language	Include
**Place of Study**	Any	Include
**Type of document**	Published Research Articles	Include
	Opinion Pieces/ Viewpoints/ Perspectives	Exclude
	Editorials / Commentaries/ Letters/ Responses	Exclude
	Reports / Case Studies / Vignettes / Anecdotal / Personal Accounts	Exclude
	Scoping / Systematic Reviews	Exclude
	Conference Abstracts	Exclude
	Meeting/ Conference Proceedings / Conference Abstracts/ Posters	Exclude
	Books/ Book Reviews	Exclude
	Grey Literature	Exclude
**Publication Status**	Not Published	Exclude
**Type of content**	Article NOT focussing on situations experienced by health and care workers unequally	Exclude

The proposed search string will be run and based on outputs of the first 100 articles, the search strategy will be adjusted for sensitivity and specificity.

### Stage 3: Study selection

Data extraction: Data outputs from the four databases will be imported into Rayyan software, duplicates will be removed using software’s automated and AI detection, and a consolidated excel file will be exported for screening and data charting. For articles that the authors are unable to retrieve with the institutional access, support will be sought from the university library team.

Title and Abstract screening: First titles and then abstracts will be screened by two reviewers to exclude studies that qualify the exclusion criteria [Table 2]. In addition to the first author, who has prior experience of HRH-related situational profiling, strategy development and research, an external relevant expert with experience of scoping reviews will be invited as a second reviewer to support with screening of articles. A small sample of scoped studies will be selected to pilot the application of eligibility criteria, and presented to the second and third authors to smooth out any disagreements and seek consensus before moving ahead with the final screening.

Text reading: Full text of included studies will be assessed for eligibility, and reasons for exclusion will be provided for studies that will be rejected. Data for the included studies will be charted.

Discrepancy assessment: Results of the two reviewers will be assessed, discrepancies will be discussed and if required, a third reviewed will be invited to make a final decision. The entire flow will be documented and presented in the PRISMA flow chart.

No critical quality appraisal will be undertaken for this scoping review, since this is not required in a scoping review and is completely optional as per JBI and PRISMA Guidelines for scoping reviews [33].

### Stage 4: Charting the data

A preliminary data charting form has been developed to determine variables to extract. The sample data extraction chart in JBI manual was used as a basic skeleton with fields added to it to serve the needs of the scoping review. The initial review iterations and subsequent discussions with the supervisory team have aided in identifying the study features, types of inequalities, experiences, workforce outcomes, methodologies etc. to be extracted [S2 Table].

Data abstraction will be conducted using the Excel output file that will be developed a priori and pilot-tested on a sample set of papers. Revisions, amendments, and additions will be made to the chart along the process to capture rich information, while simultaneously correcting the extraction of previously extracted studies. During the review process, inconsistencies in the charting will be resolved and fields may be reduced/ added to make data more meaningful. For example, multiple studies use race, culture and ethnicity interchangeably. These fields will be charted separately but at the time of descriptive analysis of the studies included, based on thematic overlaps categories may be folded/ collapsed. The data entry fields in Excel file have been designed to ensure pivoting and cross-tabulation for descriptive analysis later.

Both quantitative and qualitative data will be abstracted on study objectives, country of setting, existing frameworks or theories used to generate knowledge, types of knowledge synthesis approaches, key results, types of factors being studied (e.g. gender, race, parenthood etc.), types of experiences as a result (discrimination, prejudice, bullying, harassment etc.), the resulting impact on health and care workers’ career (barriers to career progress, leadership, penalties) or their well-being (psychosocial burnout, physical fatigue etc.), behaviours adopted by health and care workers embodying these experiences (choosing to ignoring, increasing effort, leaving work etc.). Fields like year of publication, country of publication, DOI and published keywords will be automatically extracted at the time of search and imported into the software.

### Stage 5: Collating, summarising, and reporting the results

Basic descriptive analysis will be undertaken by the first author to map the origins/ country settings of the selected papers, types of papers, inequalities and bases, health and care workforce outcomes etc. This data will be presented in tables and graphs. MS Excel and Power Bi will be used to develop charts. Snapshot summaries of the final included studies will be presented.

Although scoping review guidelines do not call for analysis beyond basic descriptive analysis, such as frequency counts of concepts, populations etc. mapped in tables or graphs [37,38], a thematic analysis will also be undertaken [39] to capture the breadth and depth of information beyond the frequency of appearing of concepts in the scoped literature, following the Braun and Clarke’s method with some modification [40,41]. No software will be used for this thematic analysis. Based on the pilot studies reviewed earlier, on average any included study in this review is expected to unpack one-three bases of inequalities and discuss on average at least two possible outcomes. Keeping this in context, the articles will not be assigned individual codes. Instead multiple codes will be assigned to each article and Maguire and Delahunt‘s approach of theoretical thematic analysis will be used to capture information that is relevant to, or of interest vis-à-vis the scoping review objective [40]. Pre-set codes will not be assigned, and instead open coding will be done, developing and modifying the codes as more articles are scoped. The initial codes [42] will be captured in the Excel output form.

Once the coding is completed, the codes will be re-examined and if found similar/related, will be grouped under a theme. For example, articles studying mental health impact on health and care workers, distress, burn-out, exhaustion may be grouped as ‘burning out’, under ‘embodiment of discriminatory experiences by health and care workers and impact on their personal well-being’. The themes will be predominantly descriptive. Overlapping themes will be re-grouped. Finally, a thematic map will be drawn to conceptualize the evidence covered and respond to the original objective of the scoping review. In addition, codes derived by these themes will also be re-assigned to the included articles to develop an evidence heat map.

The sections of analysis, discussion and conclusion of the scoping review will be co-developed by the three authors, reflecting the rich experience and subject expertise of the authors: that of inequalities in health systems, social inequalities and intersectionality, and health systems strengthening and restructuring.

## Conclusion

To the authors’ best knowledge, this review protocol is the most recent and comprehensive to report on the breadth of literature mapping the inequalities experienced by health and care workers. Its main aim is to understand the kinds of inequalities, their bases and health and care workforce outcomes covered in literature. The scoping review will reveal the different points within the HLM covered in the literature on health and care workers’ experiences, identifying gaps in knowledge for informing policies and planning, and it is hoped that its results will be relevant to policy makers as well as HRH workers.

### Dissemination plans

The results of this review will be disseminated through publications in peer-reviewed journals, conference presentations, op-eds and social media discussion threads in researcher networks working on HRH and health systems strengthening.

## Study design limitations

To authors’ knowledge and based on the literature search undertaken for this, this is the first scoping review to explore impact of multiple inequalities experienced by health and care workers on their professional and personal lives. Since a date restriction has not been set, as a consequence a broad extent of literature is expected to provide the basis for this scoping review. Furthermore, articles of interest published in other languages will be missing in this review, especially those from countries in East Asia and Europe.

## Supporting information

S1 Table Keywords developed for population and concept. (DOCX)

S2 Table Preliminary data extraction chart. (DOCX)

S1 File PRISMA scoping review checklist. (PDF)
