# Supplementary material for: Exploring the inequalities experienced by health and care workforce and their bases – A scoping review protocol
Source: PLoS One. 2024 Apr 16;19(4):e0302175. doi: 10.1371/journal.pone.0302175 (PMC11020832; doi:10.1371/journal.pone.0302175)
Supplement: S1 Table — (DOCX) [file pone.0302175.s001.docx]

**S1. Table. Keywords developed for Population and Concept**

| PCC Component | | Keywords for Scientific Databases |
| --- | --- | --- |
| Population: Health and Care Workforce | Generic Health Workers | "Health Manpower" OR "Health Human Resource" OR "Human Resource for Health" OR "Human Resources for Health" OR "Health Workforce" OR "HRH" OR "HWF" OR "Health Personnel" OR "Health Care Provider*" OR "Health* Provider*" OR "Health Care Worker*" OR "Health* Worker*" OR "Health Care Professional*" OR "Health* Professional*" OR "Health Care Practitioner*" OR "Health* Practitioner*" OR "Medical Workforce" OR "Medical Personnel" OR "Medical Professional*" |
|  | Generic Care Workers | “Social Worker*” OR “Social Care Worker*” OR “Health and Care Worker*” |
|  | Specific Health and Care Workers | OR nurs* OR dentist* OR "Dental Surgeon" OR "Dental Professional" OR doctor* OR physician* OR surgeon* OR pharmacist* OR physiotherapist* OR "Allied Health Professional*" OR midwives OR "Birth Attendant*" OR "Laboratory Staff" OR "Laboratory Personnel" OR "Paramedic*" OR "Community* Provider*" OR "Community* Worker*" OR "Health Auxiliar*" OR "Medical Auxiliar*" OR "health aide" OR "medical aide" OR "CHAP" OR "CHA/P" OR "CHP" OR "Health Extension Worker" OR "HEW" OR "Accredited Social Health Activist*" OR OR "outreach worker*" OR "field worker*" OR "health agent*" OR "surveillance assistant*" OR "health assistant*" OR "medical assistant*" OR "Health* Volunteer*" |
|  | Region Specific Cadres | "ASHA" OR "Asili" OR "MARVI" OR "Female Community Health Volunteer" OR "FCHV" OR "health* visitor*" OR "LHW*" OR "LHV*" OR "CHW*" OR "CMW*" OR "CHAS" OR "Agentes de Saúde" |
| Concept: Inequalities/ Inequities experienced by HWF |  | Equit* OR Equal* OR Inequalit* OR Inequit* OR Discrimin* OR Bias* OR Disparit* OR Injust* or Unfair* OR Prejudic* OR Stereotyp* |
