# Supplementary material for: Exploring the inequalities experienced by health and care workforce and their bases – A scoping review protocol
Source: PLoS One. 2024 Apr 16;19(4):e0302175. doi: 10.1371/journal.pone.0302175 (PMC11020832; doi:10.1371/journal.pone.0302175)
Supplement: S2 Table — (DOCX) [file pone.0302175.s002.docx]

**S2. Table. Preliminary Data Extraction Chart**

| Category | Fields |
| --- | --- |
| Name of Study | Title |
|  | Abstract |
|  | URL |
| Study Summary | Type of Content |
|  | Methodology |
|  | Pop of Interest |
|  | Research Question |
|  | Aims / Purpose of Study/ Article |
|  | Geographical Setting |
|  | Inequality of interest |
|  | Noteworthy ethical considerations |
|  | Key Findings & Recommendations |
|  | Existing Tools/ Frameworks used |
|  | Limitations |
|  | Others (More rows to be added as unpacked in the analysis) |
| Socio-economic and cultural bases of inequalities | Gender |
|  | Ethnicity |
|  | Race |
|  | Religion |
|  | Culture |
|  | Appearance |
|  | Language/ Accent |
|  | Geography |
|  | Urban-Rural |
|  | Weight |
|  | Age |
|  | Educational Background |
|  | Socioeconomic status |
|  | Sexual Orientation |
|  | Sexual Identity |
|  | Profession/ Cadre |
|  | Nationality/ Nativity |
|  | Disability |
|  | Others (More rows to be added as unpacked in the analysis) |
| Inequalities as experienced by HWF | Racism |
|  | Bullying |
|  | Verbal Harassment |
|  | Sexual Harassment |
|  | Physical Violence |
|  | Discrimination |
|  | Prejudice |
|  | Others (More rows to be added as unpacked in the analysis) |
| HWF Embodiment | Adaptation |
|  | Resilience |
|  | Increased effort |
|  | Switch place of work |
|  | Peer Support |
|  | Leave work |
|  | Ignore/Accepted status quo |
|  | Change Specialty |
|  | Not considering this Specialty |
| Impact on career | Barriers to career progression |
|  | Barriers to leadership roles |
|  | Punishments |
|  | Barriers to admission |
|  | Barriers to recruitment |
|  | Discriminatory task distribution |
|  | Compromised Learning |
|  | Missed Networking/ Mentoring Opportunities |
|  | Physical Exhaustion |
|  | Job Control |
|  | Psychological Burnout |
|  | Professional Burnout |
|  | Under-representation |
|  | Poor Performance |
|  | Additional Notes |
